# Activation of TGR5 protects blood brain barrier via the BRCA1/Sirt1 pathway after middle cerebral artery occlusion in rats

**DOI:** 10.1186/s12929-020-00656-9

**Published:** 2020-05-08

**Authors:** Hui Liang, Nate Matei, Devin W. McBride, Yang Xu, Jiping Tang, Benyan Luo, John H. Zhang

**Affiliations:** 1 Department of Neurology, First Affiliated Hospital, School of Medicine, Zhejiang University, Hangzhou, 310003 China; 2 Department of Physiology and Pharmacology and Department of Anesthesiology, Loma Linda University, 11041 Campus St, Risley Hall, Room 219, Loma Linda, CA 92354 USA; 3 The Vivian L. Smith Department of Neurosurgery, McGovern Medical School, The University of Texas Health Science Center at Houston, Houston, Texas 77030 USA

**Keywords:** TGR5, Blood-brain barrier, Neuroprotection, BRCA1, Sirt1, Middle cerebral artery occlusion

## Abstract

**Background:**

The disruption of the blood–brain barrier (BBB) plays a critical event in the pathogenesis of ischemia stroke. TGR5 is recognized as a potential target for the treatment for neurologic disorders.

**Methods:**

This study investigated the roles of TGR5 activation in attenuating BBB damage and underlying mechanisms after middle cerebral artery occlusion (MCAO). Sprague-Dawley rats were subjected to model of MCAO and TGR5 agonist, INT777, was administered intranasally. Small interfering RNA (siRNA) for TGR5 and BRCA1 were administered through intracerebroventricular injection 48 h before MCAO. Infarct volumes, brain water content, BBB permeability, neurological scores, Western blot, immunofluorescence staining and co- immunoprecipitation were evaluated.

**Results:**

Endogenous TGR5 and BRCA1 were upregulated in the injured hemisphere after MCAO and TGR5 expressed in endothelial cells. Treatment with INT777 alleviated brain water content and BBB permeability, reduced infarction volume and improved neurological scores at 24 h and 72 h after ischemia. INT777 administration increased BRCA1 and Sirt1 expression, as well as upregulated expressions of tight junction proteins. Ischemic damage induced interaction of TGR5 with BRCA1. TGR5 siRNA and BRCA1 siRNA significantly inhibited expressions of BRCA1 and Sirt1, aggravated BBB permeability and exacerbated stroke outcomes after MCAO. The protective effects of INT777 at 24 h after MCAO were also abolished by TGR5 siRNA or BRCA1 siRNA.

**Conclusions:**

Our findings demonstrate that activating TGR5 could reduce BBB breakdown and improve neurological functions through BRCA1/Sirt1 signaling pathway after MCAO. TGR5 may serve as a potential new candidate to relieve brain injury after MCAO.

## Background

Stroke is one of the most common causes of death and the main cause of long-term disability worldwide [1]. It has been recognized that disruption of the blood–brain barrier (BBB) is a critical event in the pathogenesis of stroke [2, 3]. During ischemia stroke, the disruption of BBB can lead to extravasation of solutes and fluids into the brain, resulting in vasogenic edema [4], which causes a poor clinical outcome. Therefore, the pharmacological targeting of protecting BBB may be a promising treatment strategy for cerebral infarction [5].

TGR5 is a plasma membrane-bound G protein-coupled bile acid receptor, which is present in various tissues, including in animal and human brain [6, 7]. TGR5 is recognized as a potential target for the treatment for hepatic disorders, metabolic disorders, and kidney disease, through anti-inflammation, anti-apoptosis and inhibition of oxidative stress [8, 9]. In *central nervous system* (CNS), studies have found that activating TGR5 alleviates brain damage and improves outcomes in a model of experimental autoimmune encephalomyelitis (EAE) and hepatic encephalopathy [10, 11]. Nevertheless, the effects of TGR5 on BBB integrity in brain injuries after ischemic stroke have not been investigated.

BRCA1, a tumor suppressor gene implicated in breast and ovarian cancers, is expressed by endothelial cells and can improve endothelial dysfunction, which may provide a protective role in neurological diseases [12–14]. Several researches have established that BRCA1 is a key regulator of sirtuin 1 (Sirt1) [15, 16]. Sirt1 is a nicotinamide adenine dinucleotide-dependent deacetylase, which is involved in the regulation of physiological functions, including cell senescence, gene transcription, energy balance, and oxidative stress. Studies have confirmed the protective role of sirt1 against BBB damage in CNS pathologies [17, 18].

In the present study, we hypothesized that (1) activating TGR5 protects BBB damage and attenuates brain insult after middle cerebral artery occlusion (MCAO) and (2) the protection of TGR5 on the BBB is mediated through a BRCA1/Sirt1-related signaling pathway.

## Materials and methods

### Animals

All experiments were approved by the Institutional Animal Care and Use Committee of Loma Linda University (approval no. 8170034) and Zhejiang University (approval no. 2016–193). All animal care and use were conducted according to the Guide for the Care and Use of Laboratory Animals (National Research Council). All procedures of experiments are reported in compliance with the ARRIVE (Animal Research: Reporting in Vivo Experiments) guidelines. Animals were housed in a 12 h light-dark cycle, temperature-controlled room. A total of 494 Sprague-Dawley male rats (2–3 months, weighing 250–300 g) were used in the study.

### MCAO model

The transient MCAO model was induced as previously described [19]. Rats were anesthetized intraperitoneally with a mixture of ketamine (80 mg/kg) and xylazine (20 mg/kg). Briefly, the right common carotid artery (CCA), internal carotid artery (ICA) and external carotid artery (ECA) were surgically exposed. 4–0 nylon suture with silicon was inserted into the ICA through the ECA stump until the tip of the suture reached the origin of the anterior cerebral artery (ACA) (approximately 18 to 22 mm). After 2 h of occlusion, the suture was withdrawn to allow for reperfusion. During surgery, body temperature was maintained at a physiological level. Sham groups underwent the same procedure but without occluding the MCA.

#### Experimental design

**Fig. 1:**
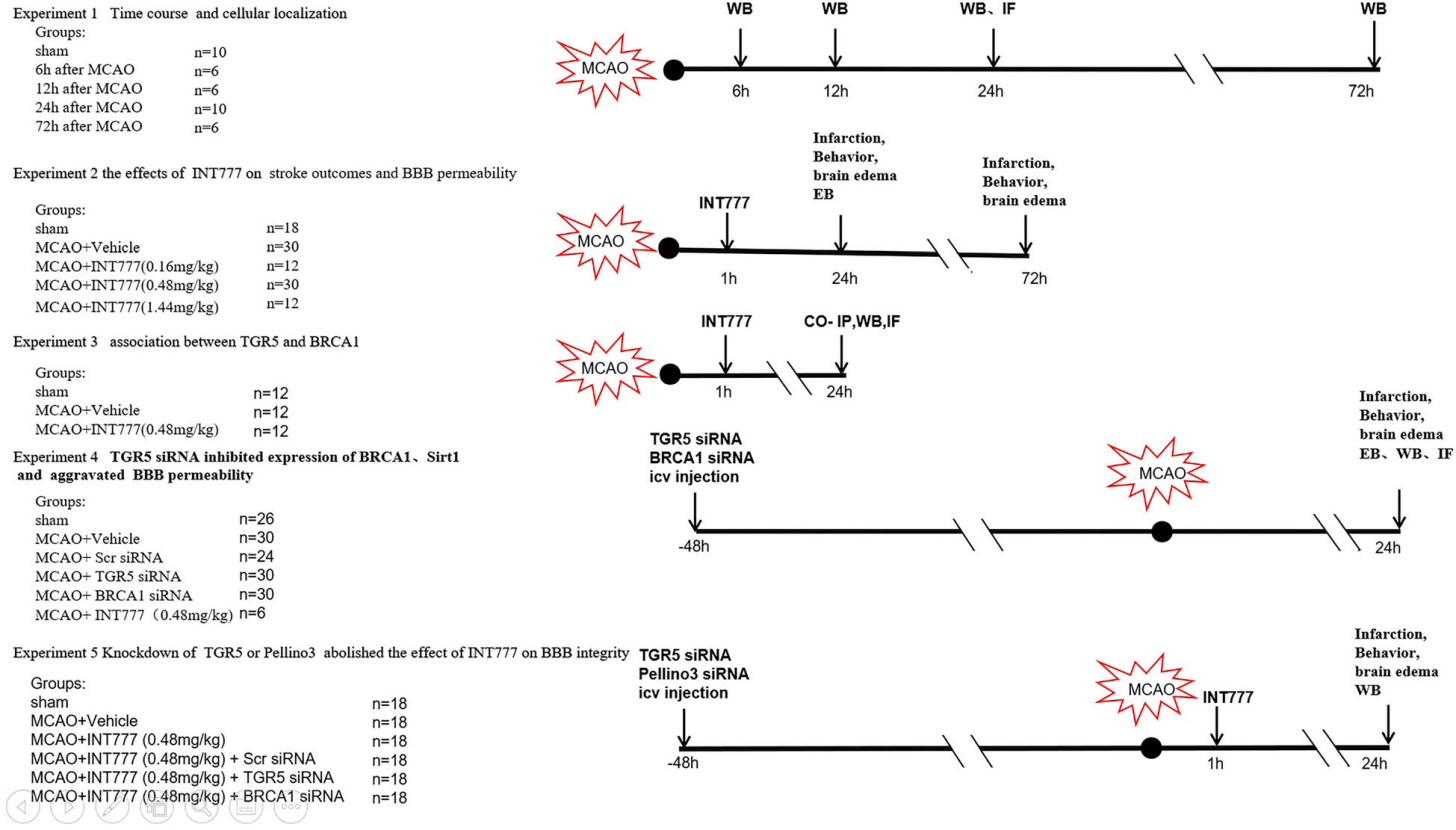
Experimental design and animal group classification. IF, immunofluorescence; icv, intracerebral ventricular; MCAO, middle cerebral artery occlusion; Scr siRNA, Scramble small interfering RNA; WB, Western blot; Co-IP, Co- immunoprecipitation

A schematic diagram of our research design was shown in Fig. 1.

##### Experiment 1

The time course of endogenous expressions of TGR5 and BRCA1 in right hemispheric tissue was evaluated by Western blot.30 rats were divided into 5 groups: Sham (*n* = 6), MCAO 6 h (*n* = 6), MCAO 12 h (*n* = 6), MCAO 24 h (*n* = 6), and MCAO 72 h (*n* = 6). An additional 8 rats, sham (*n* = 4) and MCAO 24 h (*n* = 4), were used for immunofluorescence staining to characterize the localization of TGR5 in endothelial cells (visualized using an antibody against von Willebrand factor (VWF)).

##### Experiment 2

One hundred two rats were used in the following groups: sham (*n* = 18), MCAO+vehicle (*n* = 30), MCAO+INT777 (0.16 mg/kg, *n* = 12), MCAO+INT777 (0.48 mg/kg, *n* = 30), MCAO+INT777 (1.44 mg/kg, *n* = 12). Infarction volume, neurobehavior scores, and brain water content were measured at 24 and 72 h after MCAO. Evans blue (EB) extravasation was evaluated at 24 h after MCAO and barrier function assessment in vitro was assessed by TEER. Based on neurological tests at 24 h and 72 h after MCAO, the middle dosage of INT777 (0.48 mg/kg) was chosen for further studies.

##### Experiment3

Thirty-six rats were divided into 3 groups for exploring the association between TGR5 and BRCA1 by co-immunoprecipitation: sham (*n* = 12), MCAO+vehicle (*n* = 12), MCAO+INT777 (*n* = 12). The immunofluorescence staining samples for co-labeling of TGR5 with BRCA1 were shared with experiment 1.

##### Experiment 4

To explore the effect of knocking down TGR5 and BRCA1 on stroke, 146 rats were randomly assigned to the following 5 groups: Sham (*n* = 26), MCAO+vehicle (*n* = 30), MCAO+Scramble siRNA (*n* = 24), MCAO+TGR5 siRNA (*n* = 30), MCAO+BRCA1siRNA (*n* = 30), MCAO++INT777 (*n* = 6). Infarction volume, neurobehavior scores, brain water content, EB extravasation, immunofluorescence staining and Western blots were measured. Four samples of sham for immunofluorescence staining were shared with experiment 1.

##### Experiment 5

One hundred eight rats were randomly assigned to 6 groups for mechanism study: sham (*n* = 18), MCAO+vehicle (*n* = 18), MCAO+INT777 (*n* = 18), MCAO+INT777 + scramble siRNA (*n* = 18), MCAO+INT777 + TGR5 siRNA (*n* = 18), and MCAO+INT777 + BRCA1 siRNA (*n* = 18). Neurobehavioral scores, brain infarction, brain water content and Western blot were evaluated.

### Drug administration

Intranasal administration of INT777 (MedChemExpress,USA) was performed as previously described [20], with some modifications: rats were administered either saline, INT777 (0.16 mg/kg), INT777 (0.48 mg/kg) or INT777 (1.44 mg/kg) intranasally (5 μL/drop) over a period of 20 mins, alternating drops every 2 min between left and right nares. The total volume delivered was 50 μL at 1 h following MCAO.

### Intracerebroventricular siRNA injection

Three different formats of TGR5-siRNA or BRCA1-siRNA (OriGene Technologies) were diluted with transfection reagent (entranser™,Engreen Biosystem) and were injected 48 h before MCAO by intracerebroventricular injection (ICV) as previously described [21, 22]. The ICV injection site was relative to location of bregma: anteroposterior 1 mm, right lateral 1.5 mm, depth 3.5 mm. The TGR5-siRNA, BRCA1-siRNA mixture or scramble-siRNA (100 pmol in 5 μL) was delivered into the ipsilateral ventricle with a Hamilton syringe (Microliter 701, Hamilton Company, Reno, NV) and administered over 5 min. The needle was left for 5 min after injection and was then slowly withdrawn over 5 min. After the needle was removed, the burr hole was sealed with bone wax.

### Neurological scores

Neurobehavioral outcomes were assessed by a blinded investigator at 24 h and 72 h following MCAO [23]. The sensorimotor function scores were evaluated as following: spontaneous activity, symmetry in limb movement, symmetry of forelimb outstretching, climbing, body proprioception, response to vibrissae touch, and beam walking. The neurological scoring ranged from 3 (most severe deficits) to 21 (normal).

#### 2.6. 2,3,5-Triphenyltetrazolium chloride (TTC) staining

Infarction volume was evaluated by TTC staining after MCAO [24]. standard methods were used to correct the possible interference of brain edema on infarct volume and infarction volume was expressed as a ratio of the whole brain volume, as previous report [25]*.*

### Brain water content

Brains were separated into left hemisphere, right hemisphere, cerebellum, and brain stem at 24 or 72 h after MCAO. Each brain samples were weighed immediately after removal (wet weight) and then dried in an oven at 105 °C for 72 h (dry weight). The percentage of brain water content was calculated as [(wet weight-dry weight)/wet weight] Χ100% [23].

### BBB permeability

BBB permeability was evaluated by EB extravasation using spectrophotometry as previously described [26]. At 4 h before sacrifice, 2% EB dye in saline was injected intravenously as a BBB permeability tracer. A microplate fluorescence reader was used to determine EB dye fluorescence intensity. The amount of extravasated EB dye was quantified as micrograms per ischemic hemisphere.

### Barrier function assessment of in vitro

The in vitro experiment was prepared as previously reported [27], with some modifications.bEnd.3 cells were purchased from the Bioleaf Biotech Co., Ltd. (Shanghai, China) and were cultured as previously described. After the bEnd.3 cells were treated with INT777 (30 μmol/l), OGD/R was induced in the cells for 6 h in a hypoxia chamber in RPMI 1640 culture medium without glucose, in an atmosphere of1% O2, 5% CO2, and 94% N2.Then the cells were cultured under normoxia conditions in normal culture medium after 2 h of OGD for 18 h. The integrity ofthe bEnd.3 cell monolayer was measured via the TEER assay.

### Immunofluorescent staining

The method of double and triple immunofluorescence staining was performed as previously described [28, 29]. Rats were transcardially perfused with cold phosphate-buffered solution (PBS) followed by 10% paraformaldehyde after rats were deeply anesthetized at 24 h after MCAO. The whole brains were fixed in 10% paraformaldehyde for 24 h then in 30% sucrose solution for 72 h. Coronal frozen slices (10 μm) were obtained with a cryostat (CM3050S; Leica Microsystems, Wetzlar, Germany) and permeabilized with 0.3% Triton X-100 in PBS for 30 min. Sections were blocked with 5% donkey serum for 1 h and incubated at 4 °C overnight with primary antibodies: anti-TGR5 (1:100 Abcam), anti-BRCA1 (1:100 Santa Cruz Biotechnology), anti- vWF (1:100 Abcam) and anti- CD31 (1:100 Abcam). The slices were viewed with fluorescence microscope (DMi8; Leica Microsystems, Germany) or confocal LSM 710 microscope and fluorescence intensity was quantified using ImageJ.

### Western blot analysis

Western blot analysis was performed as previously reported [30]. Proteins of the ipsilateral hemisphere were extracted by homogenizing in radio-immunoprecipitation assay lysis buffer. Equal amounts of a sample protein were loaded onto an SDS-PAGE gel. First, electrophoresis and transfer of the samples to a nitrocellulose membrane were performed. Second, the membrane was blocked for 2 h at room temperature and incubated overnight at 4 °C with the following primary antibodies: anti-TGR5 (1:1000, Abcam), anti-BRCA1 (1:1000, Santa Cruz Biotechnology), anti-Sirt1(1:1000, Abcam), anti-occludin (1:2000, Abcam, USA), anti-ZO-1(1:200, Santa Cruz Biotechnology) and anti-β-actin (1:5000, Santa Cruz Biotechnology). The secondary antibodies were all from Santa Cruz Biotechnology. Blot bands were visualized with an ECL reagent (Amersham Biosciences UK Ltd., PA, USA) and were quantified by densitometry using Image J software (Image J 1.4, NIH, USA).

### Co-Immunoprecipitation (co-IP)

Co-IP was performed as previously described [20, 31].500 μg protein incubated with TGR5 antibody (1:50) or BRCA1 antibody (1:50) and agitated. Protein A/G agarose (20 μL; Sigma) was added to each sample and incubated overnight at 4 °C. Next, the mixture was precipitated by high-speed freezing centrifugation at 12000 rpm for 10 s. Then the sediment was washed three times with NP-40 buffer. Agarose-bound immunocomplexes were released using a denaturing solution. TGR5 and BRCA1 proteins in immunocomplex denaturing solution and total protein solution (for comparison) were analyzed by Western blot.

### Statistical analysis

All data analyses were performed using SigmaPlot 11.0 and GraphPad Prism 6 (GraphPad software, San Diego, CA). Parametric data was expressed as mean ± SEM. Data from different groups were compared using one-way ANOVA followed by post hoc Tukey tests. Non-parametric data (neurological scores, beam walking) were analyzed with the Kruskal–Wallis test followed by Dunn’s post-hoc. In all statistical analysis, *P* < 0.05 was considered as significant.

## Results

### Mortality and exclusion

A total of 494 rats were used and 410 rats underwent MCAO induction. There were no deaths in sham group. For groups of MCAO, the mortality rate was 11.5% (47 of 410) (Supplementary Table 1). Seventeen animals were excluded if rats didn’t show signs of neurobehavioral deficits when waking up from MCAO (body twisting when lifted by the tail and walking in circles) or if subarachnoid hemorrhage was found during euthanasia.

### Endogenous TGR5 receptor and BRCA1 expression increased after MCAO

**Fig. 2:**
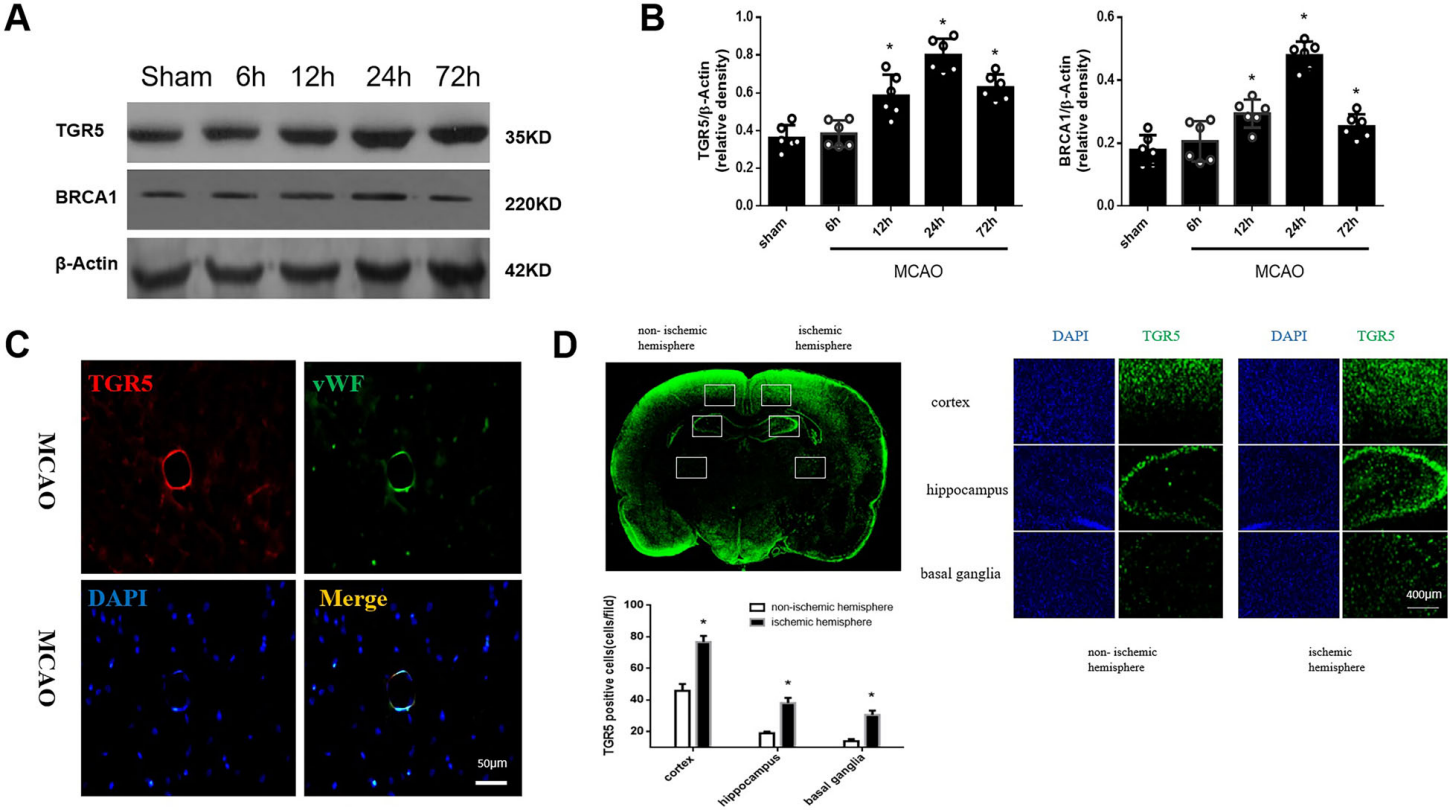
Expression of TGR5 and BRCA1 in the right hemisphere of the rat brain after MCAO. **a**, **b** Representative Western blot images and quantitative analyses of TGR5 and BRCA1 time-course expression after MCAO. *n* = 6 per group. **c** Double immunofluorescence staining revealed that both TGR5 (red) was predominantly expressed in vWF positive- endothelial cells (green) in penumbra at 24 h after MCAO. *n* = 4 per group. *P < 0.05 vs sham group. Bars represent mean ± SEM. Scale bar, 50 μm. vWF, Von Willebrand factor. **d** Whole brain immunofluorescence staining showed that TGR5 expression was upregulated in the cortex, hippocampus and basal ganglia 24 h after MCAO. *n* = 4 per group. *P < 0.05 vs contralateral non-ischemic hemisphere. Bars represent mean ± SEM. Scale bar, 400 μm

We investigated TGR5 and BRCA1 alterations after MCAO. In Fig. 2a and b, TGR5 and BRCA1 expressions significantly increased from12 hours to a peak at 24 h but declined at 72 h after MCAO (*P* < 0.05 versus Sham). Double immuno-fluorescence staining demonstrated that TGR5 was expressed in endothelial cells at 24 h after MCAO (Fig. 2c). Whole brain immunofluorescence staining showed that TGR5 expression was upregulated in the cortex, hippocampus and basal ganglia 24 h after MCAO when compared with contralateral non-ischemic hemisphere (Fig. 2d).

### INT777 improved stroke outcomes and BBB permeability after MCAO

**Fig. 3:**
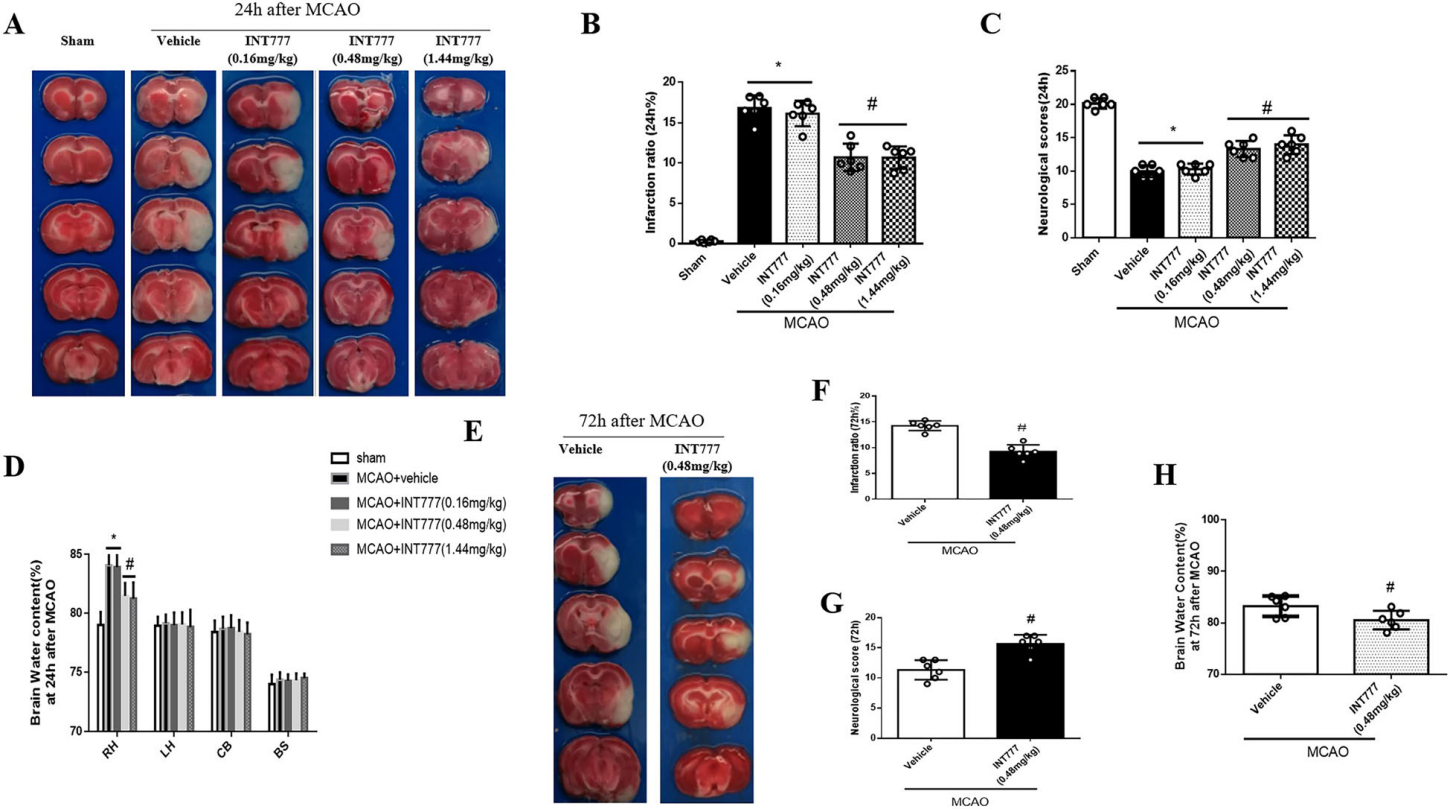
Exogenous TGR5 agonist INT777 ameliorated brain injury at 24 h and 72 h after MCAO. Representative TTC staining indicated brain infarction at 24 h and 72 h after MCAO (**a**, **e**);quantified infarct ratio(**b**, **f**), neurological scores (**c**, **g**) and brain water content(**d**, **h**) showed that INT777 decreased infarction and neurological deficits in medium dose as well as reduced brain edema of right ischemic hemisphere at 24 h and 72 h after MCAO . High dose was only analyzed at 24 h after MCAO. *n* = 6 for each group. *P < 0.05 vs sham, ^#^*P* < 0.05 vs MCAO+ vehicle. Bars represent mean ± SEM. BS indicates brain stem; CB, cerebellum; LH, left hemisphere; RH, right hemisphere

Treatment with 0.48 mg/kg and 1.44 mg/kg of INT777 significantly reduced infarct volume, improved neurological scores and reduced brain water content of right ischemic hemisphere at 24 h post-MCAO compared to MCAO+vehicle group (Fig. 3a-d) (*P* < 0.05). The administration of 0.48 mg/kg INT777 decreased cerebral infarction, restored neurological function and ameliorated brain water content at 72 h after injury (Fig. 3e-h) (*P* < 0.05 versus MCAO+vehicle). Based on the dose study, we chose to middle dosage of INT777 for all subsequent studies.

**Fig. 4:**
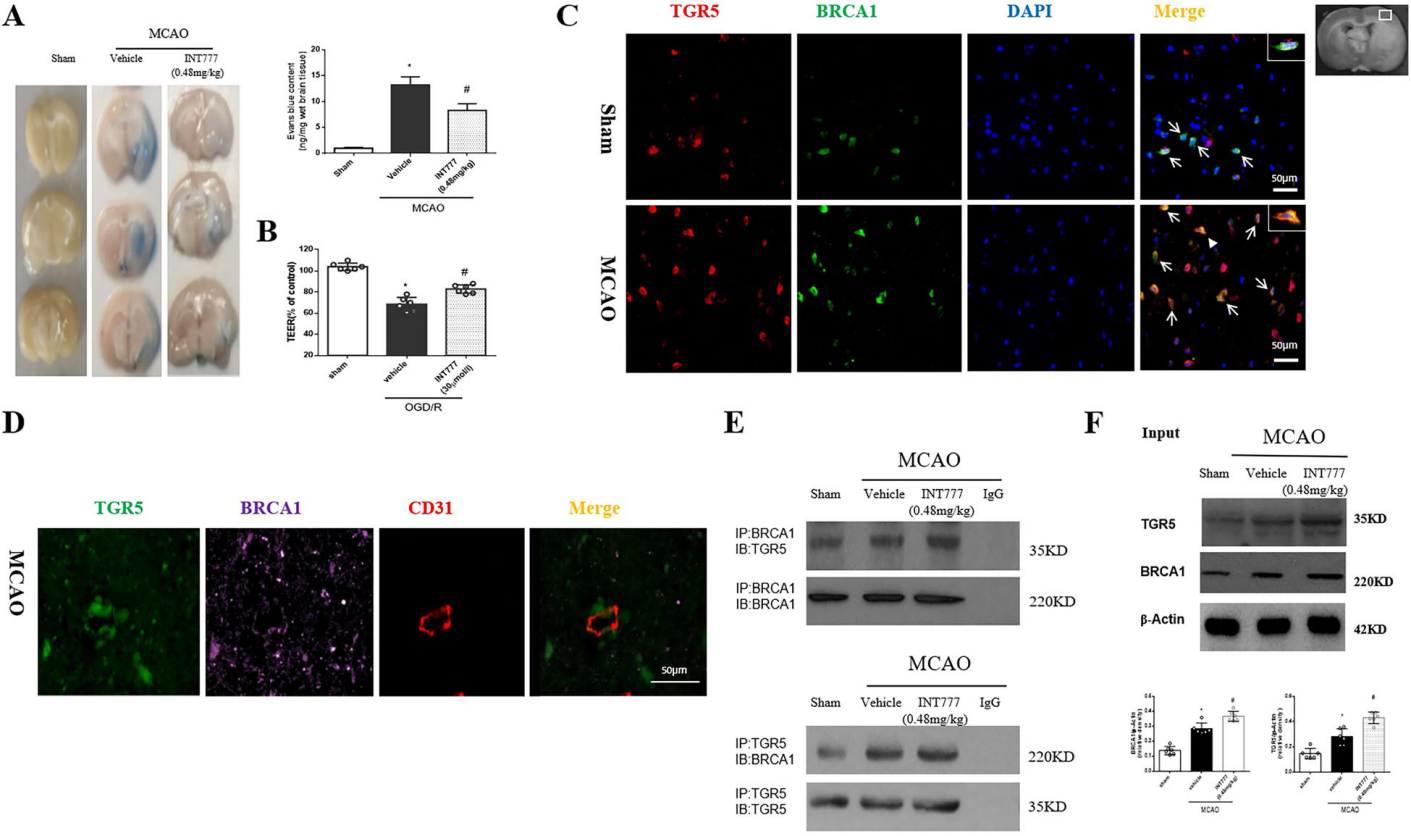
INT777improved BBB permeability and TGR5 interacted with BRCA1 after MCAO. **a** INT777 treatment significantly reduced EB dye leakage, *n* = 6 per group; **b** INT777 alleviated the TEER decrease after OGD/R, *n* = 6 per group; **c** Double immunofluorescence staining showed that co-localization of TGR5 (red) and BRCA1 (green) was increased in penumbra 24 h after MCAO, **d** Triple-fluorescence staining showed that TGR5 and BRCA1colocalized in endothelial cell, *n* = 4 per group. Scale bar 50 μm. **e** Representative co-IP bands showed that interactions of TGR5 with BRCA1 occurred at 24 h after MCAO, *n* = 6 per group. **f** Expression of TGR5 and BRCA1 in total protein solution was detected by Western blot analysis and relative OD ratios were reported. *n* = 6 for each group. *P < 0.05 vs sham, ^#^*P* < 0.05 vs MCAO+ vehicle. Bars represent mean ± SEM

EB extravasation was markedly increased at 24 h post-MCAO (*P* < 0.05 versus sham), INT777 treatment significantly reduced EB dye leakage(*P* < 0.05 versus MCAO+vehicle) (Fig. 4a). INT777 also alleviated the TEER decrease in the in vitro model after OGD/R(*P* < 0.05 versus OGD/R + vehicle) (Fig. 4b).

### MCAO induced interactions between TGR5 and BRCA1

In the sham group, double immunofluorescence staining showed that co-labeling of TGR5 with BRCA1 was detected in the brain. After ischemic injury, co-labeling of TGR5 with BRCA1 increased in the penumbra area (Fig. 4c). Triple-fluorescence staining also showed that TGR5 and BRCA1 co-localized in endothelial cell (Fig. 4d). Western blot showed that both TGR5 and BRCA1 expression increased at 24 h after MCAO (*P* < 0.05 versus sham), and INT777 further increased TGR5 and BRCA1 expression(*P* < 0.05 versus MCAO) (Fig. 4f). CO-IP showed that TGR5- BRCA1 interaction was found in the ischemic hemisphere (Fig. 4e).

### TGR5 siRNA inhibited expression of BRCA1/Sirt1 and aggravated BBB permeability after MCAO

**Fig. 5:**
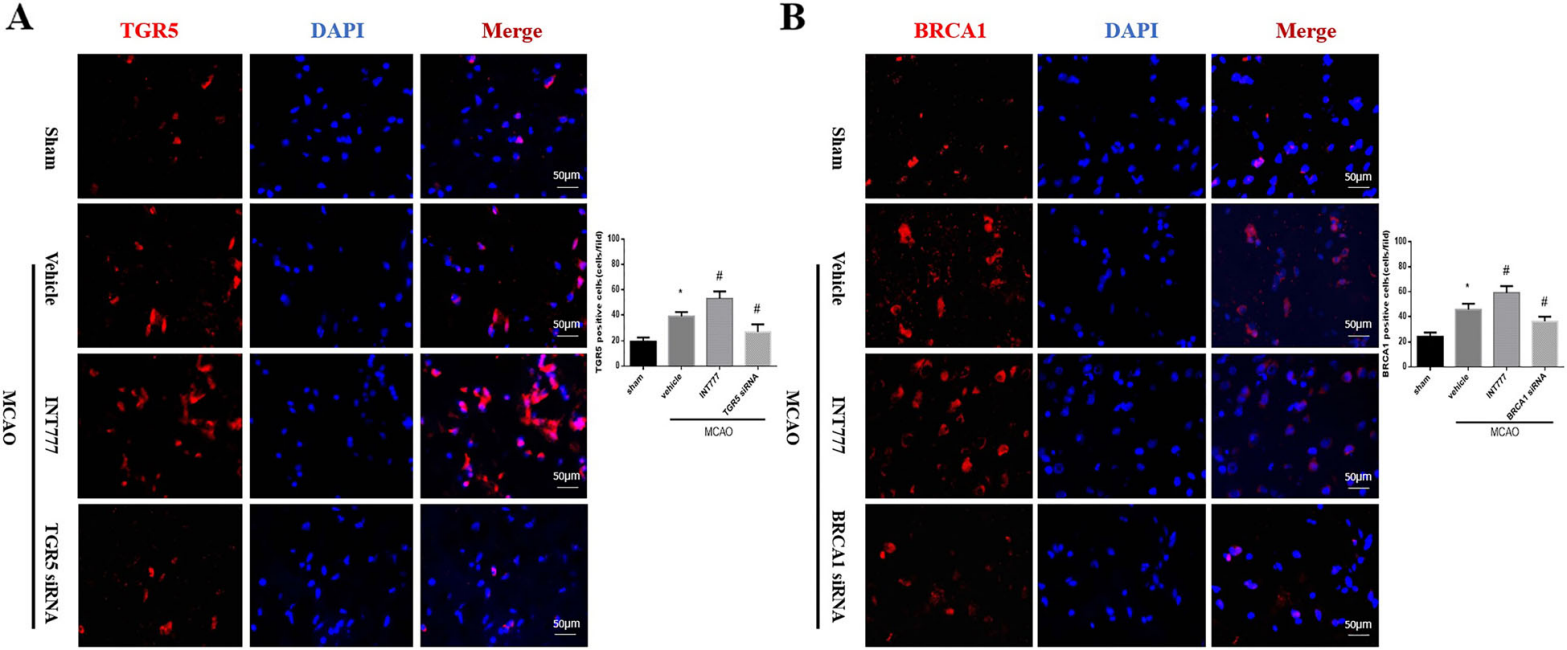
The effect of INT777 and siRNA on TGR5 and BRCA1 expression in penumbra after middle cerebral artery occlusion (MCAO). **a**, **b** Double immunofluorescence staining showed that expression of TGR5 or BRCA1 was upregulated in the penumbra area 24 h after MCAO; INT777 treatment increased the expressions while siRNA inhibited TGR5 or BRCA1 expression. *n* = 6 per group. *P < 0.05 vs sham, ^#^*P* < 0.05 vs MCAO+ vehicle. Bars represent mean ± SEM. Scale bar, 50 μm

To further assess the role of TGR5 in stroke, TGR5 siRNA was administered by ICV injection to knockdown endogenous TGR5. Double immunofluorescence staining showed that both TGR5 and BRCA1 expressions increased in penumbra following MCAO while siRNA significantly reduced TGR5 or BRCA1 expression (Fig. 5a and b).

**Fig. 6:**
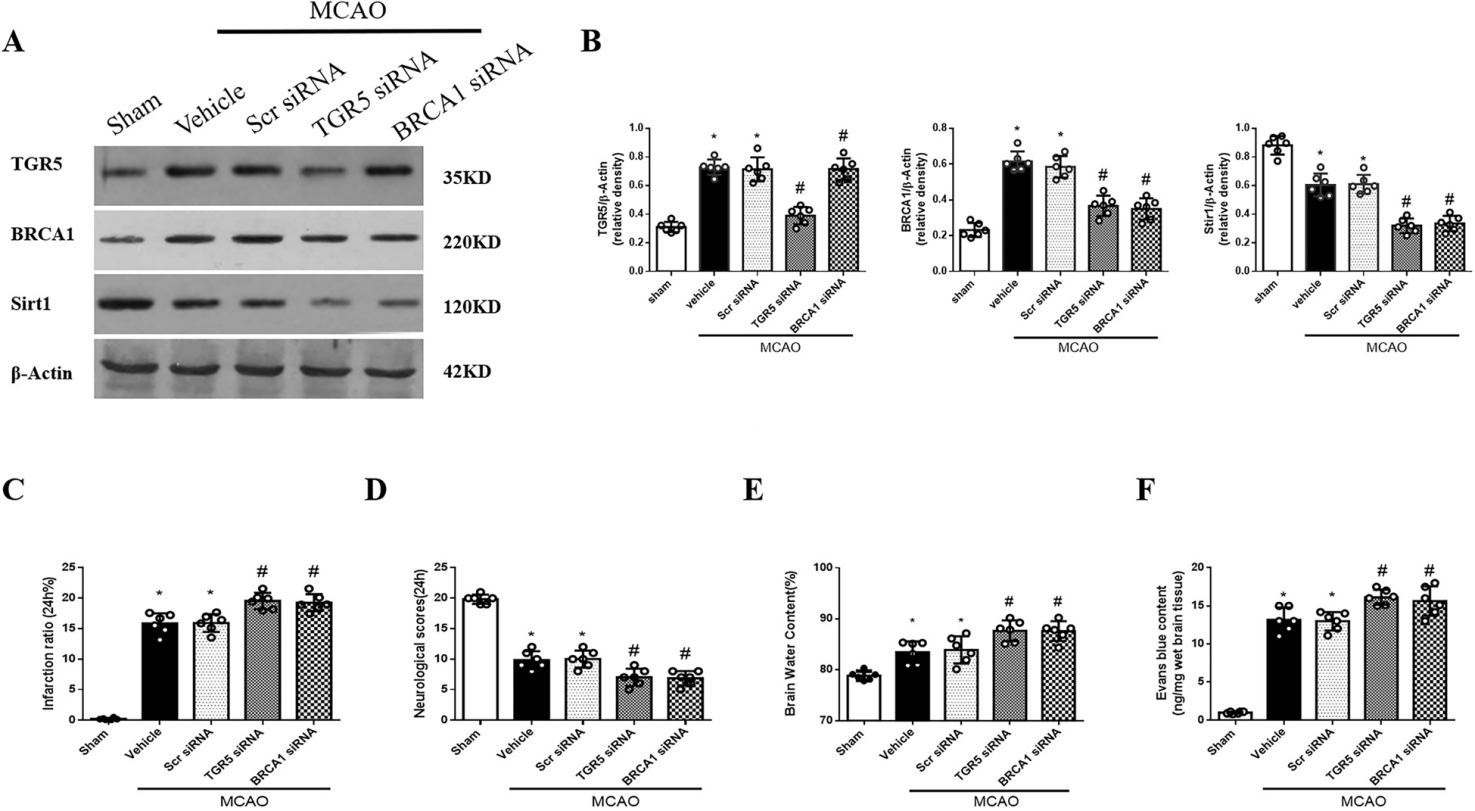
The effect of knockdown TGR5 on BRCA1/Sirt1 expression and BBB permeability after MCAO. **a** The band of Western blot analysis; **b** The relative density of TGR5, BRCA1, Sirt1.*n* = 6 per group. TGR5 or BRCA1 knockout increased infarct volume (**c**), worsen neurobehavioral deficits (**d**), exacerbated brain water content (**e**) and BBB permeability (**f**). *n* = 6 per group. *P < 0.05 vs sham, ^#^*P* < 0.05 vs MCAO+ Scr siRNA. Bars represent mean ± SEM. Scr siRNA, scramble siRNA

The results of Western blot staining showed that TGR5 expression was partially prevented by TGR5 siRNA (Fig. 6a and b). Compared with scramble siRNA group, TGR5 siRNA significantly inhibited expressions of BRCA1 and Sirt1 after MCAO (*P* < 0.05) (Fig. 6a and b). The knockdown efficacy of BRCA1 siRNA was also confirmed by Western blot and BRCA1 knockdown markedly decreased the Sirt1 expression and had no effect on TGR5 expression after MCAO (Fig. 6a and b).

Both TGR5 siRNA or BRCA1 siRNA significantly exacerbated stroke outcomes and aggravated BBB permeability after MCAO (*P* < 0.05) (Fig. 6c-f), when compared with scramble siRNA group at 24 h after MCAO.

### TGR5 or BRCA1 knockdown abolished the protective effects of INT777 on BBB integrity after MCAO

**Fig. 7:**
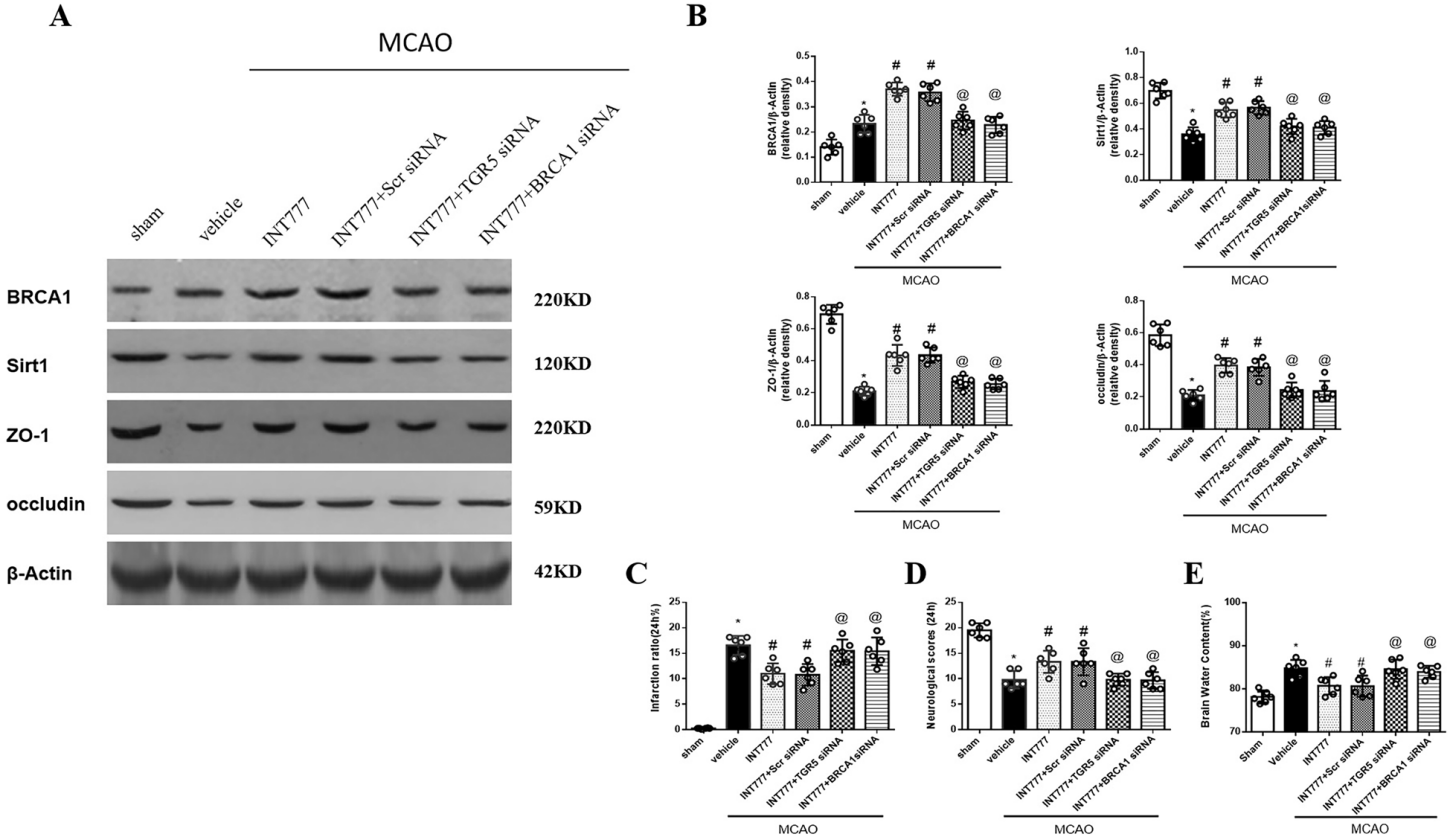
Knockdown TGR5 or BRCA1 abolished the protective effects of INT777 on BBB permeability after MCAO. **a** Representative Western blots. **b** Quantitative analyses of TGR5, BRCA1, Sirt1, zo-1, occludin. Quantified infarct ratio (**c**), neurological scores (**d**) and brain water content (**e**), *n* = 6 per group. *P < 0.05 vs sham, ^#^*P* < 0.05 vs MCAO+ vehicle,^@^*P* < 0.05 vs MCAO+INT777 + Scr siRNA group. Scr siRNA, scramble siRNA

Decreased tight junction (TJ) protein expressions or variations are associated with alterations in BBB permeability [32]. As shown in Fig. 7a and b, Sirt1 and TJ proteins (ZO-1 and occludin) were remarkably decreased at 24 h after SAH, when compared with the sham group (*P* < 0.05). However, INT777 increased expressions of Sirt1, ZO-1 and occludin, compared with MCAO group (*P* < 0.05) (Fig. 7a and b). The results above demonstrated that INT777 alleviated disrupted BBB by increasing TJ proteins in ischemic brain.

When compared with INT777+ scramble siRNA group, TGR5 siRNA reversed the effect of INT777 on the expressions of TGR5, BRCA1, Sirt1, ZO-1and occludin at 24 h after MCAO (*P* < 0.05) (Fig. 7a and b). Western blot showed that BRCA1 siRNA also abolished the effects of INT777, leading to reduce expressions of Sirt1, ZO-1 and occludin (*P* < 0.05) (Fig. 7a and b).

Administration of TGR5 siRNA or BRCA1 siRNA significantly abolished the protective effect of INT777 on infarction volume, neurological deficits and brain edema at 24 h after MCAO (*P* < 0.05 versus MCAO+INT777 + Scramble siRNA) (Fig. 7c-e).

## Discussion

In the present study, we first described the TGR5 mediated signaling pathway in BBB protection after MCAO in rats. Our data demonstrated that TGR5 and its essential downstream protein BRCA1 were upregulated in the injured hemisphere after MCAO. Exogenous TGR5 agonist INT777 reduced brain edema and BBB permeability and thereby alleviated stroke outcome after MCAO. In contrast, knockdown of endogenous TGR5 or BRCA1 by siRNA exacerbated brain edema, BBB disruption, infarction volume, and neurological deficits. INT777 increased TGR5, BRCA1 and Sirt1 expressions, as well as upregulated TJs. Furthermore, knockdown TGR5 or BRCA1 by siRNA abolished the beneficial effects of INT777, which were associated with reduced Sirt1, ZO-1 and occludin. Taking together, our study suggested that activating TGR5 may be involved in regulating BBB permeability after MCAO at least in part via a BRCA1 /Sirt1 signaling pathway.

Research have showed that Bile acids, such as tauroursodeoxycholic acid (TUDCA), play an important role of neuroprotection for brain pathologies which are mediated by TGR5 [33]. McMillin et al. found that TGR5 is present in the cortex of C57Bl/6 mice and is upregulated in the brain following azoxymethane induced acute liver failure. This up-regulation appears to be protective, as activating TGR5 reduces neurological decline [11]. In the model of EAE, mice treated with TGR5 agonists had significant reductions in the clinical score both at peak of disease and at the termination of the study [10]. In the present research, we observed that TRG5 was upregulated in the penumbra after MCAO and was expressed in endothelial cells. The administration of INT777 significantly diminished BBB disruption and improved stroke outcomes after MCAO, whereas silencing endogenous TGR5 by siRNA aggravated BBB breakdown and neurological deficits.

Although the exact mechanisms of TGR5–mediated BBB protection are not well clarified, BRCA1 may play an important role in the TGR5-mediated signaling pathway. BRCA1, a well-known tumor suppressor implicated in familial breast and ovarian cancers, provides a protective role in atherosclerosis and neurological diseases [12–14]. Noristani et al. found that BRCA1 is expressed by human microglia and is dysregulated in humans and an animal model of ALS [34]. Several studies demonstrated that BRCA1 deficiency contributes to neuronal injury in Huntington’s Disease and impairs cognitive function in mice [13, 14]. In vitro experiments, two major bile acids, deoxycholic acid and chenodeoxycholic acid, were found to increase BRCA1 expression relative to untreated control OVCAR3 ovarian cancer cells, through interaction with bile acid receptors [35]. In the present study, we observed that endogenous BRCA1 expression was increased at 24 h after MCAO and INT777 further augmented BRCA1 expression. Double immunofluorescence staining demonstrated an increased co-localization of TGR5 with BRCA1 after MCAO, and using CO-IP, we found an interaction between TGR5 and BRCA1 after MCAO. Furthermore, we observed that silencing TGR5 inhibited the expression of BRCA1 and reversed the protective effect of INT777 on BRCA1 expression. Taken together, these findings support that TGR5 is upstream to activate BRCA1, thereby alleviating BBB damage.

Several evidence have confirmed that BRCA1 is a key regulator of Sirt1 in cancer research. BRCA1 inactivation events (mutation, promoter methylation, or knockdown) are accompanied by decreased Sirt1 levels while overexpression of BRCA1 results in increased Sirt1 levels [15] by direction binding of BRCA1 to the Sirt1 promoter [16]. More studies showed that Sirt1 plays a major role in protecting against brain injuries during ischemia stroke [36]. Chen et al. reported that the activation of Sirt1 was associated with increased BBB permeability in vitro [37]. However, in most studies, increasing the Sirt1 level would benefit BBB damage after oxygen glucose deprivation, subarachnoid hemorrhage or sepsis-induced brain injury [17, 18, 38]. Our lab also found that Sirt1 was a key mediator of Hyperbaric Oxygen (HBO) protective effects in BBB damage after MCAO. Knockdown Sirt1 by Sirt1 siRNA reversed the protective effects of HBO [23].

In the current study, we found that INT777 increased the expression of Sirt1 after MCAO while TGR5 siRNA and BRCA1 siRNA inhibited the Sirt1 expression, reversed the effect of INT777 on Sirt1, which means TGR5 and BRCA1 can act as upstream regulators of Sirt1. Furthermore, our data demonstrated that TGR5 or BRCA1 knockdown significantly reverses the neuro-protection of INT777 on stroke outcomes, as well as decreasing ZO-1 and occludin expression. This finding supports the notion that the BRCA1/ Sirt1 signaling pathway plays a role in BBB protection induced by activation of TGR5 after MCAO.

There are some limitations in the present study. First, TGR5 produces pleiotropic effects via different signaling pathways, such as alleviating inflammation and attenuating apoptosis [39]. In this study, we only focused on the neuroprotective effects of TGR5 on BBB integrity after MCAO, but further studies are needed to explore other effects of TGR5 after MCAO and its underlying signaling mechanisms. Second, only young male rats were used. Following the STAIR recommendations, we need to repeat the key findings using aged males, as well as female rats.

## Conclusions

**Fig. 8:**
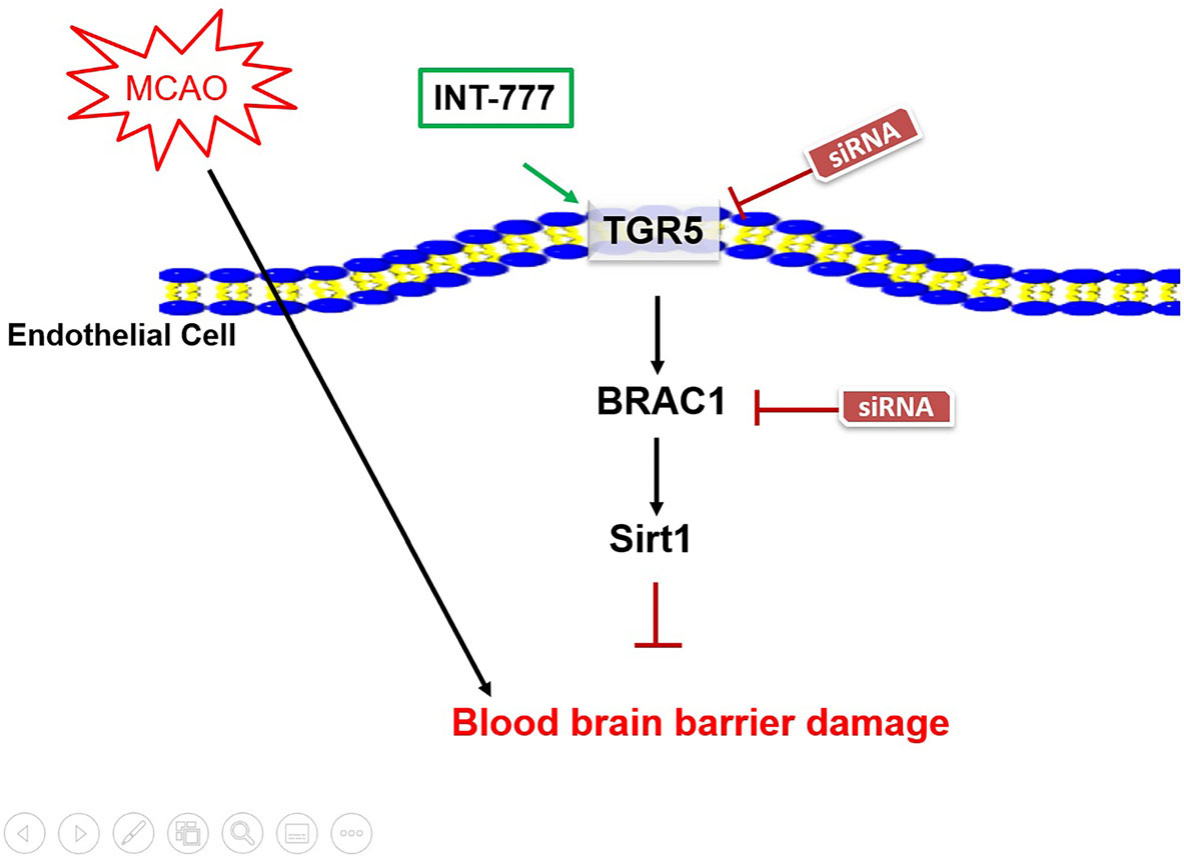
Proposed pathway in the present study. This study found that TGR5 agonist, INT777, could protect BBB and improve neurological outcomes after MCAO, which through BRCA1/Sirt1 signaling pathway after MCAO. Our findings suggest that TGR5 may serve as a potential new candidate to relieve brain injury after MCAO

As summarized in Fig. 8, we found that activating TGR5 could reduce BBB breakdown and improve neurological deficits after ischemic stroke. The results highlight TGR5/BRCA1/Sirt1 signaling as a critical contributor to alleviate BBB damage and as a novel target for brain edema in diseases characterized by BBB damage, such as stroke, inflammatory diseases, and neurodegenerative diseases.

## Supplementary information

**Additional file 1.**

## Data Availability

All data used during the current study available from the corresponding author on reasonable request.

## Abbreviations

ACA: Anterior cerebral artery
BBB: Blood-brain barrier
CCA: Common carotid artery
CNS: *Central nervous system*
Co-IP: Co-Immunoprecipitation
*EAE*: Experimental autoimmune encephalomyelitis
ECA: External carotid artery
ICA: Internal carotid artery
MCAO: Middle cerebral artery occlusion
PBS: Phosphate-buffered solution
siRNA: Small interfering RNA
TJ: Tight junction
TTC: Triphenyltetrazolium chloride
TUDCA: Tauroursodeoxycholic acid
