# Correction to: Activation of TGR5 protects blood brain barrier via the BRCA1/Sirt1 pathway after middle cerebral artery occlusion in rats

**DOI:** 10.1186/s12929-020-00666-7

**Published:** 2020-06-03

**Authors:** Hui Liang, Nathanael Matei, Devin W. McBride, Yang Xu, Jiping Tang, Benyan Luo, John H. Zhang

**Affiliations:** 1 Department of Neurology, First Affiliated Hospital, School of Medicine, Zhejiang University, Hangzhou, 310003 China; 2 Department of Physiology and Pharmacology and Department of Anesthesiology, Loma Linda University, 11041 Campus St, Risley Hall, Room 219, Loma Linda, CA 92354 USA; 3 The Vivian L. Smith Department of Neurosurgery, McGovern Medical School, The University of Texas Health Science Center at Houston, Houston, TX 77030 USA

**Correction to: J Biomed Sci (2020) 27:61**

**https://doi.org/10.1186/s12929-020-00656-9**

In the original publication of this article [1] the name of second author is incorrect, the correct author name is Nathanael Matei. The error in this Correction has been updated in the original article.
