# Supplementary material for: Activation of TGR5 protects blood brain barrier via the BRCA1/Sirt1 pathway after middle cerebral artery occlusion in rats
Source: J Biomed Sci. 2020 May 8;27:61. doi: 10.1186/s12929-020-00656-9 (PMC7206796; doi:10.1186/s12929-020-00656-9)
Supplement: Supplementary file 1 — Additional file 1. [file 12929_2020_656_MOESM1_ESM.docx]

# ONLINE SUPPLEMENT Full title: Activation of TGR5 protects blood brain barrier via the BRCA1/Sirt1 pathway after middle cerebral artery occlusion in rats

# Authors’ names: Hui Liang^a,b^, Nate Matei^a^，Devin W. McBride^a,c^,Yang Xu^a^,Zhenhua Zhou^a^, Jiping Tang^a^, Benyan Luo ^b^,John H. Zhang^a^

**Table** 1. Animal number (Survival/total) in each group.

| **Group** | **TTC**  **（24h）** | **TTC**  **（72h）** | **Brain water**  **content** | **EB** | **WB** | **IF** | **Time**  **course** | **CO-IP** |
| --- | --- | --- | --- | --- | --- | --- | --- | --- |
| sham | 18/18 |  | 18/18 | 12/12 | 18/18 | 6/6 | 6/6 | 6/6 |
| MCAO |  |  |  |  |  | 4/4 | 24/27 |  |
| MCAO+Vehicle | 18/20 | 6/7 | 24/27 | 12/13 | 18/20 | 6/7 |  | 6/7 |
| MCAO+INT777(0.16 mg/kg) | 6/7 |  | 6/6 |  |  |  |  |  |
| MCAO+INT777(0.48 mg/kg) | 12/13 | 6/7 | 18/21 | 6/6 | 12/14 | 6/7 |  | 6/6 |
| MCAO+INT777(1.44 mg/kg) | 6/6 |  | 6/7 |  |  |  |  |  |
| MCAO+Scr siRNA | 6/7 |  | 6/7 | 6/8 | 6/7 |  |  |  |
| MCAO+TGR5 siRNA | 6/7 |  | 6/7 | 6/8 | 6/6 | 6/7 |  |  |
| MCAO+BRCA1 siRNA | 6/7 |  | 6/8 | 6/7 | 6/7 | 6/7 |  |  |
| MCAO+INT777+Scr siRNA | 6/6 |  | 6/7 |  | 6/7 |  |  |  |
| MCAO+INT777+TGR5 siRNA | 6/7 |  | 6/6 |  | 6/8 |  |  |  |
| MCAO+INT777+BRCA1 siRNA | 6/6 |  | 6/7 |  | 6/7 |  |  |  |
| Total | 96/104 | 12/14 | 108/121 | 48/54 | 84/94 | 34/38 | 30/33 | 18/19 |

**WB: western blot;IP: immunoprecipitation;** **EB：Evan's blue ; 17 excluded animals were not added in this table**
